# In vivo long-term investigation of tumor bearing mKate2 by an in-house fluorescence molecular imaging system

**DOI:** 10.1186/s12938-018-0615-0

**Published:** 2018-12-29

**Authors:** Kedi Zhou, Yichen Ding, Ivan Vuletic, Yonglu Tian, Jun Li, Jinghao Liu, Yixing Huang, Hongfang Sun, Changhui Li, Qiushi Ren, Yanye Lu

**Affiliations:** 1 Department of Biomedical Engineering, College of Engineering, Peking University, No. 5 Yiheyuan Road, Beijing, 100871 China; 2 Laboratory Animal Centre, Peking University, No. 5 Yiheyuan Road, Beijing, 100871 China

**Keywords:** mKate2, Luciferase, Molecular imaging, Tumor cell tagging

## Abstract

**Background:**

Optical imaging is one of the most common, low-cost imaging tools used for investigating the tumor biological behavior in vivo. This study explores the feasibility and sensitivity of a near infrared fluorescent protein mKate2 for a long-term non-invasive tumor imaging in BALB/c nude mice, by using a low-power optical imaging system.

**Methods:**

In this study, breast cancer cell line MDA-MB-435s expressing mKate2 and MDA-MB-231 expressing a dual reporter gene firefly luciferase (fLuc)-GFP were used as cell models. Tumor cells were implanted in different animal body compartments including subcutaneous, abdominal and deep tissue area and closely monitored in real-time. A simple and low-power optical imaging system was set up to image both fluorescence and bioluminescence in live animals.

**Results:**

The presence of malignant tissue was further confirmed by histopathological assay. Considering its lower exposure time and no need of substrate injection, mKate2 is considered a superior choice for subcutaneous imaging compared with fLuc. On the contrary, fLuc has shown to be a better option when monitoring the tumor in a diffusive area such as abdominal cavity. Furthermore, both reporter genes have shown good stability and sensitivity for deep tissue imaging, i.e. tumor within the liver. In addition, fLuc has shown to be an excellent method for detecting tumor cells in the lung.

**Conclusions:**

The combination of mKate2 and fLuc offers a superior choice for long-term non-invasive real-time investigation of tumor biological behavior in vivo.

## Background

Over the past decade, whole-body molecular imaging has become an unequivocally promising method in all pre-clinical practice and basic studies in the field of oncology [1]. Compared with other functional modalities such as positron emission tomography (PET), and single photon emission computed tomography (SPECT), optical molecular imaging has no radiation and keeps frequent tumor monitoring safe [2, 3]. In addition, optical imaging is one of the most inexpensive and rapid ways to track specific molecular targets [4]. However, in order to visualize deeper tissues, which are not visible by conventional microscopes, optical modalities have to overcome strong scattering and absorption.

Application of luminescence proteins for in vivo tumor imaging has been under investigation for decades [5, 6]. Over the past few years, various fluorescent and bioluminescent proteins with different emission spectra have been discovered [7]. Compared to fluorescent dyes or quantum dots that are commonly used to target a specific moiety, fluorescent and bioluminescence reporter genes can be stably integrated in the cell genome and specifically engineered to express a fusion protein of interest [8]. Among fluorescent proteins, red-shifted proteins are considered a superior choice for in vivo imaging because of their reduced autofluorescence, less diffusion and deeper penetration of light in the far-red and near-infrared region (NIR). A fluorescent protein named mKate2, a monomeric far-red fluorescent tag that is threefold brighter than mKate and is tenfold brighter than mPlum, is currently one of the best far-red florescence proteins available on the market [9–11]. The emission wavelength peak of mKate2 is 633 nm within the far-red and NIR optical window, while the excitation peak is 588 nm [12]. Compared with other commercial fluorescent proteins, both excitation and emission spectra of mKate2 are relatively non-absorbed and less scattered by the surrounding tissues and proteins (largely by hemoglobin) [13, 14]. Moreover, besides to the high-brightness and far-red emission spectrum, its excellent pH resistance and photostability make mKate2 a superior candidate for oncological study in live animals such as mice [15].

Unlike fluorescent proteins, bioluminescence imaging does not require an excitation light that consequently leads to significantly lower background for imaging. Compared to fluorescent proteins, their high sensitivity in live tissues is the key advantage when using firefly bioluminescent Luc [16]. Although, long exposure time, frequent substrate injection and unstable Luc signal preclude its intravital applications, bioluminescence imaging is still required where sensitivity is as critical [17, 18]. Furthermore, some researchers have applied a dual Luc-GFP fusion vector for circulating tumor in vivo studies; while Luc have high sensitivity and is excellent in vivo indicator, GFP can be used for in vitro microscopy or ex vivo imaging [19, 20]. In short, both fluorescence and bioluminescence are excellent choices for monitoring the tumor biological behavior [21, 22] and tumor therapy [23] in live animal tissues such as mice.

From the imaging system prospective, in the optical molecular imaging field [24, 25], bioluminescence tomography (BLT) [26, 27], fluorescence molecular tomography (FMT) [28, 29] and fluorescence protein tomography (FPT) [30, 31], have been extensively reported. These methods have been progressively advancing and greatly promoting the development of medicine and biological science. Nevertheless, more effectively long-term investigations of animal studies are still eagerly awaited [32, 33]. While the 3-dimensional whole-body imaging of in vivo organisms is feasible, the image reconstruction can potentially be inefficient and inaccurate and therefore the epi-illumination planar imaging allows for rapid initial screening. Considering the varying parameters of pH, temperature and concentration of oxygen in living organisms [34–36], we designed a low-power system to reduce the optical interference of normal biological process during the fluorescence imaging in vivo. Due to a high-sensitivity CCD camera and the high-brightness proteins, the light power of excitation for fluorescent proteins could be decreased to 8 mW at most from 400 to 800 nm in the whole imaging field. For the bioluminescence imaging, the system was easily converted to a light-free platform by blocking the excitation. The new stem designs can eliminate the cost of imaging system setup compared with commercial counterparts, and offers flexibility for users to adjust this system in order to meet their own needs.

In this work, we have achieved three objectives: firstly, we have set up a simple and low-power optical imaging system (LP-OIS) for both fluorescence and bioluminescence imaging of live animals; secondly, we have tested the performance of mKate2 fluorescent proteins in mice under the interference of optical diffusion and absorption; thirdly, we established an ideal strategy for in vivo long-term investigation of tumor by applying distinct luminescence reporter genes. To date, this is the first study that explores the application of mKate2, a novel far-red fluorescent protein with its higher brightness and photostability, for real-time detection of tumor in whole-body, including subcutaneous, abdominal and deep tissue compartment.

## Methods

### Low-power optical imaging system (LP-OIS)

**Fig. 1:**
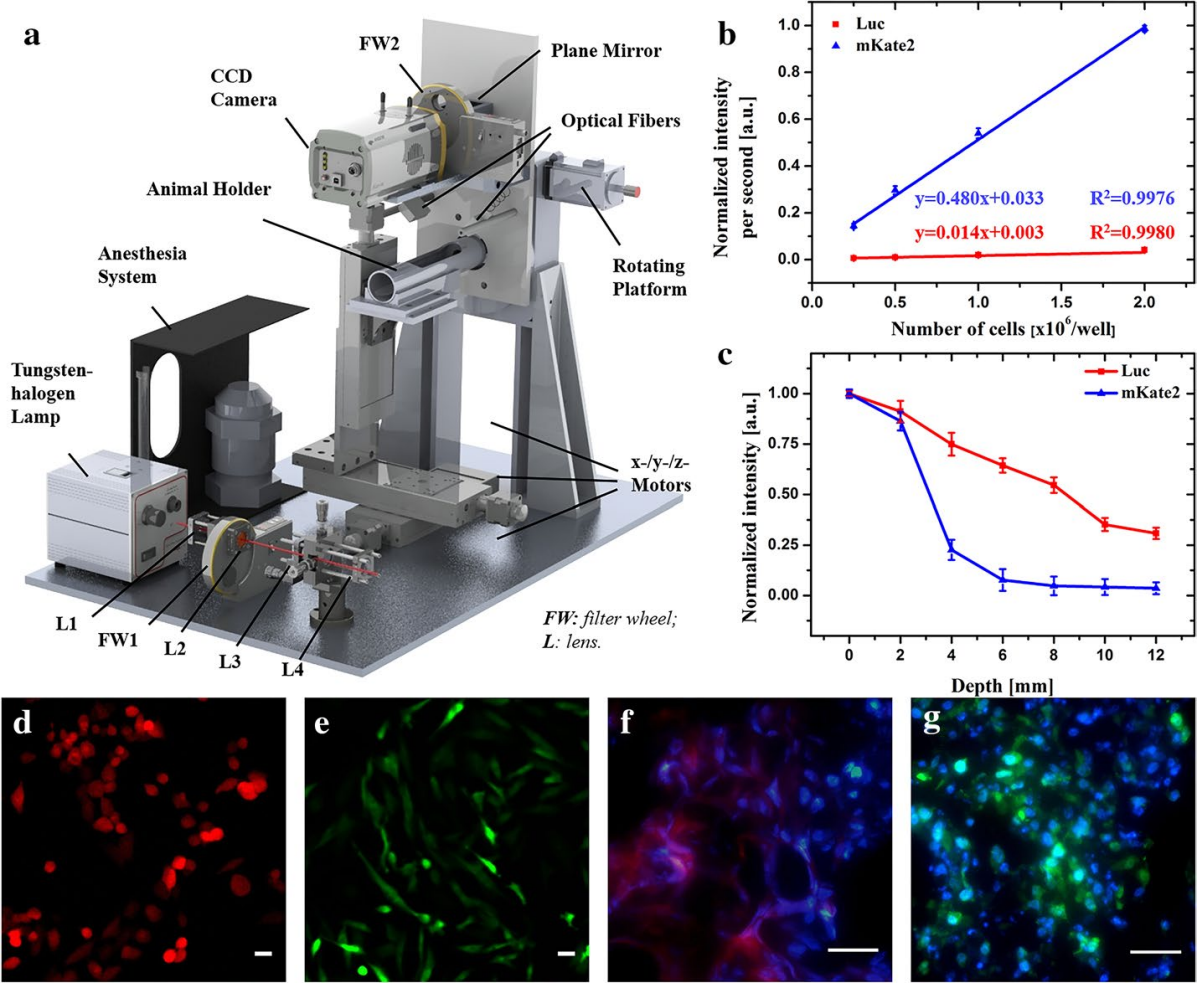
Schematic illustration and in vitro sensitivity of the whole-body optical imaging system. **a** System setup. *L* lens, *FW* filter wheel. **b** Linearity and sensitivity within 1 s and **c** imaging depth of the optical system. mKate2 and Luc are indicated in red and blue, respectively. Microscopic results of MDA-MB-435s-mKate2 cells (**d**) and tumor tissue (**f**); and MDA-MB-231-GFP cells (**e**) and tumor tissue (**g**)

The whole imaging system was composed of four main parts (Fig. 1a): a high-sensitivity CCD camera-based detection module, a tungsten-halogen lamp based excitation module, an adjustable animal holder and a gas anesthesia component.

The back illuminated, deep depletion CCD camera with fringe suppression (iKon-M 934, 16bit, Andor, UK) was critical for both fluorescence and bioluminescence imaging in this work. Its superb specifications meet the need of the LP-OIS. Firstly, the quantum efficiency of the back illuminated CCD was over 90% in the visible and NIR spectra. Roughly, in this work, the counts number could be treated quantitatively close to the induced photon flux. Secondly, its dark current was only 0.017 e^−^/pixel/s when cooling down to − 80 °C in this work. To balance the sensitivity and resolution of the image, the camera was binned 2 × 2 and therefore the ideal dark current was converted to 0.068 counts/s. Thirdly, when the read rates were set at 3 MHz, the readout noise was 9.2 e^−^ root mean square for the entire system. All the parameters were the nominal value indicated in the manual sheet and were validated again before installation in the imaging system. To acquire the emission, a 35 mm F1.4 lens (LM35HC, Kowa, Japan), with focusing range of 0.3 m to infinity was employed. The focal length of 35 mm was proper to image the whole body of a mouse on the CCD chip with readable resolution, and the large numerical aperture allows substantial photons to reach the CCD detector during exposure time. An electrical filter wheel (FW102C-EC, Thorlabs, USA) were placed in front of the lens in order to filter proper emission wavelength.

For the bioluminescence imaging, there was no need of applying external excitation light. By contrast, for the fluorescence imaging, a tungsten-halogen lamp (71LT250/71PT250A, Beijing 7-Star, China) was utilized. As Tungsten-halogen lamp provides a broad spectrum covering visible and infrared regions, we can easily use various filters for different fluorophores. It was an incandescent illumination source, generating a continuous distribution of light across the visible and NIR spectra. Under normal operating conditions, the temporal and spatial output fluctuation of this lamp was minimal and disregarded for this study. In Fig. 1a, after passing through a quartz len, an adapter and the lens to reshape the illumination, the light was filtered (BrightLine, Semrock, USA) into appropriate wavelengths for targeting probes. The beam was coupled into optical fiber bundles (G5.7-2-4000-K, G5.7-4000, Nanjing Chunhui, China) for illumination. Compared to a previous study [22], in this work, the power of illumination on the surface of experimental mouse was 8 mW at most, ranging from 400 nm to nearly 800 nm. The power was measured at its central wavelength with the bandpass filters whose bandwidth were tens of nanometers. The fiber for epi-illumination was split into two heads from both top sides, which covered a whole body of a nude mouse, with a uniform illumination in the 75 × 75 mm^2^ area.

All mice and all the in vitro experiments were handled using epi-illumination. To meet different needs of applications, when three stepper motors worked, the fiber heads were fixed in the animal holder so that they remained at the same relative positions over time. No matter imaging of fluorescence or bioluminescence, the animal was narcotized with a gas anesthesia system (VIP 3000, Matrx, USA), and both the animal holder and detection module were kept in an optical enclosure to prevent from the stray light.

### Reagents and cell lines

Dulbecco’s modified Eagle’s medium (DMEM), Fetal bovine serum (FBS), Phosphate buffered saline (PBS), Trypsin–EDTA and Lipofectamine-2000 were purchased from Invitrogen. Penicillin/streptomycin was from HyClone. G418 was bought from MP Biomedicals and pmKate2-N vector (FP182#) was from Evrogen.

The breast cancer cell line MDA-MB-435s was obtained from American Type Culture Collection (ATCC). MDA-MB-231 expressing a dual reporter Luc-GFP was a kind gift from Prof. Tian’s group (Institute of Automation, Chinese Academy of Sciences, Beijing). Both cells were cultured in DMEM supplemented with 10% FBS and 1% Penicillin/streptomycin in a humidified atmosphere containing 5% CO_2_/95% air at 37 °C.

### Construction of mKate2-expressing tumor cell line

Lipofectamine 2000 reagent was used for cell transfection. 3.5 × 10^5^ cells were plated per well in a 6-well plate. When cells reached 90–95% confluence during 24 h incubation, the culture medium with antibiotics was replaced with a new one without antibiotics and serum; in the meantime, 2 μg plasmid DNA pmKate2-N and 6 μL Lipofectamine-2000 were diluted in 0.25 mL medium without antibiotics and serum in separate tubes, and consequently mixed and incubated for 20 min at room temperature. A total of 0.5 mL of the reagent was then added in each well and swirled to ensure even distribution. The G418 selection was initiated 24 h post transfection by adding predefined concentration of 800 μg/mL G418 in the culture medium.

### Testing the optical system sensitivity in vitro

For linearity testing, MDA-MB-435s-mKate2 cells and MDA-MB-231-Luc-GFP cells were harvested, dispersed into a single cell suspension and seeded in with different concentration in a 96-well plate. For imaging cells in different depths, 2 × 10^6^ cells were immersed in the 1% intralipid solution. We imbedded the cells in 1% intralipid solution at different depths as the phantom study. This in vitro study enabled us to assess the imaging performance at different depths in the homogeneous solution. All conditions of the imaging system were the same as in vivo experiments.

### Animals

BALB/c female nude mice, 6- to 8-week-old, weighing 20–25 g, were obtained from Beijing Vital River Laboratory Animal Technology Co., China. All the animals were housed in an environment with temperature of 24 ± 1 °C, relative humidity of 50 ± 1% and a light/dark cycle of 12/12 h. All mice were given tap water and special Alfalfa free laboratory rodent chow [37]. Before the experiment, mice were fastened for 12 h.

All animal studies (including the mice euthanasia procedure) were done in compliance with the regulations and guidelines of Peking University Institutional Animal Care and conducted according to the AAALAC and the IACUC guidelines.

### Tumor implantation in vivo

For in vivo imaging studies, MDA-MB-435s-mKate2and MDA-MB-231-Luc-GFP were carefully implanted in different mice body compartments using the following methods.

**Fig. 2:**
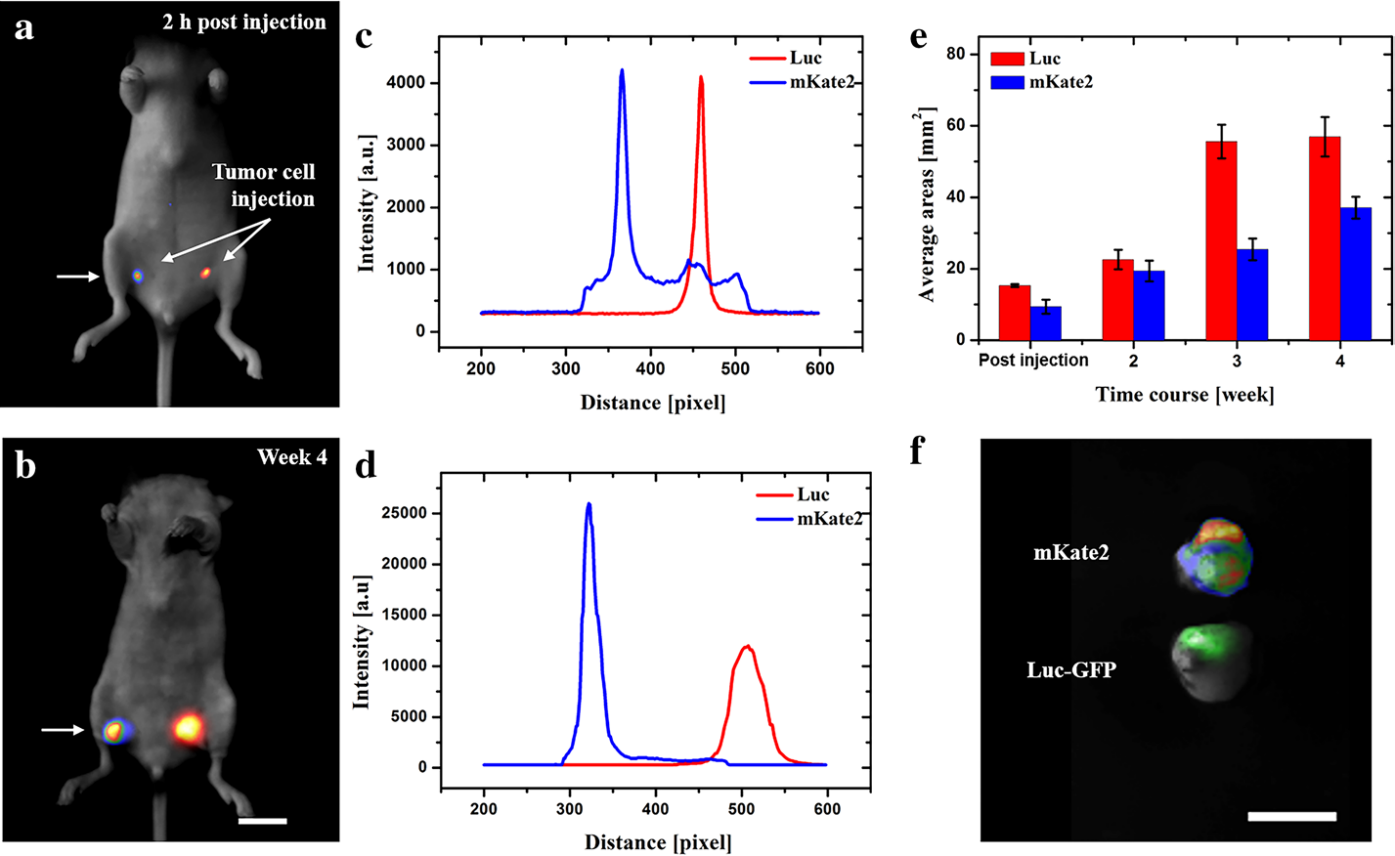
In vivo fluorescence and bioluminescence imaging of subcutaneous MDA-MB-435s-mKate2 and MDA-MB-231-Luc-GFP tumor. **a**, **b** In vivo images of tumor expressing different reporter genes in different time points. **c**, **d** Intensity profiles are corresponding to the areas along the arrow axis. **e** Real-time quantification of total tumor-luminescence surface area (mm^2^) detected over 4 weeks post-cells injection. **f** Ex vivo results of genetic tagging tumors labeled by mKate2 (rainbow) and GFP (green). Scale bar: 10 mm
- *Abdominal tumor implantation* Tumor cells were trypsinized and washed three times with fresh PBS. Tumor dissemination in the abdominal cavity was obtained by direct injection of 2 × 10^6^ MDA-MB-435s-mKate2 (200 μL, cells mixed with PBS) in the peritoneal region by using a 1 mL 27G latex-free syringe within 30 min of harvesting (abdominal injection location indicated in Fig. 3a).

- *Subcutaneous tumor implantation* All cells were harvested by trypsinization and washed three times with fresh PBS. After collecting of cells, 1 × 10^6^ cells of each cell line were injected in the lower right flank and lower left flank of the same nude mice, respectively (subcutaneous injection location indicated in Fig. 2a).

**Fig. 3:**
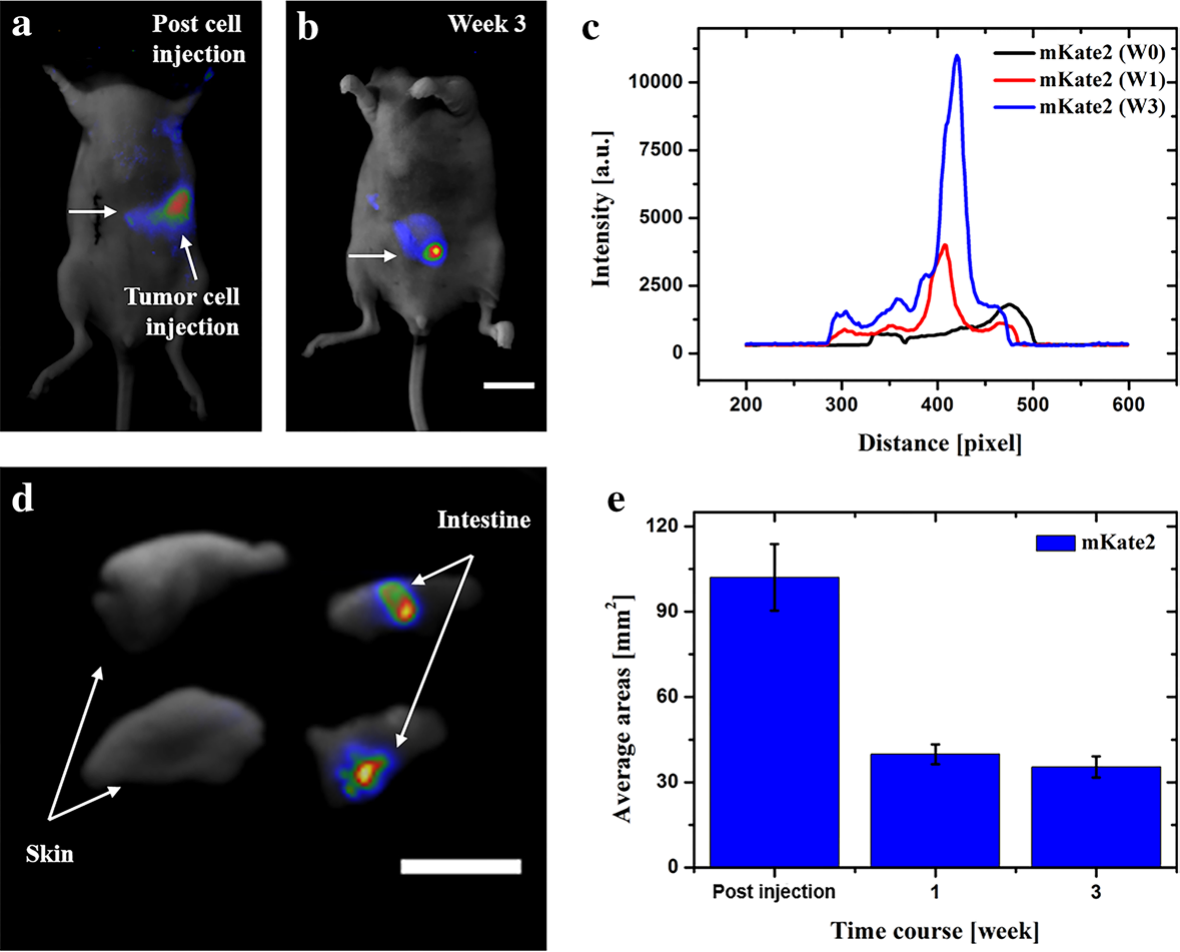
In vivo fluorescence of abdominal MDA-MB-435s-mKate2 tumor development. **a**, **b** In vivo images of tumor locations in the abdominal cavity. **c** Intensity profiles are corresponding to the areas along the arrow axis. Plot profiles of mKate2 through different weeks are indicated in black, red and blue, respectively. **d** Ex vivo tissue examination postmortem. **e** Real-time quantification of total tumor-fluorescence surface area (mm^2^) detected over 3 weeks post-cells injection. Scale bar: 10 mm
- *Deep tissue tumor implantation* The *t*umor infections of the main organs were investigated by three protocols. First, 5 × 10^6^ MDA-MB-435s-mKate2 cells were implanted directly in the liver (liver injection location indicated in Fig. 5a). Cells were injected with a total volume of 50 μL into a nude mouse, by using a 1 mL 29G, latex-free syringe within 30 min of harvesting the cells. After cell implantation, the injection site was compressed for 1 min with a cotton swab to prevent the leakage of tumor cells out of the liver. The abdominal wall and the skin were then closed using 6-0 surgical suture. Secondly, in order to test the spontaneous cell invasion of abdominal cavity, MDA-MB-435s-Luc-GFP cells were first subcutaneously implanted under the lower left flank of a nude mouse. After the tumor has reached 10 mm in diameter, the tumor tissue was carefully dissected, washed with PBS and cut with sterile scissors in small pieces of 1 mm^3^. The small tumor block was then carefully implanted under the left upper liver lobe membrane of a nude mouse. The implanted site was then cleaned with a cotton swab. The abdominal wall and the skin were then closed using 6-0 surgical suture. Thirdly, 2 × 10^6^ of mKate2 expressing tumor cell and Luc-GFP expressing tumor cell were injected in a total volume of 200 μL into the lateral tail vein of different nude mice respectively.

### Data processing

We performed single-shot acquisition for all images. Based on our preliminary test of mice (n = 6) bearing an MDA-MB-435s-mKate2 tumor, the intensity of autofluorescence excited by 588 nm light was around 1000 counts. Thus, in the following in vivo studies the effective fluorescent intensity of infected regions was set empirically above 1000 counts. The maximum intensity and area of infected region could be analyzed in real time during imaging.

For the MDA-MB-435s-mKate2 cells, 1 s exposure time was used for all experiments. For the MDA-MB-231-Luc-GFP cells, d-luciferin was injected intraperitoneally, and the bioluminescence signal was acquired 15 min post d-luciferin injection. The exposure time of bioluminescence was 30 s in the subcutaneous and in vitro cases, while the time 300 s was used for the abdominal and organs (lung and liver) detection cases. Furthermore, GFP was used for detection of MDA-MB-231 tumor in ex vivo and in vitro (excluding the testing for the sensitivity and linearity of the optical imaging system) tests. Also, the raw data were acquired under the control of baseline clamp and processed using Andor SOLIS, and therefore the background was not related to exposure time any more. The maximum intensity and the area of the infected regions were calculated using a plugin of Andor SOLIS. The brightness and contrast of images were processed using ImageJ. The curves and linear fitting were recalculated using Origin 8.0.

## Results

### Cell sensitivity in vitro and ex vivo

The in vitro experimental results of tumor expressing mKate2 and tumor expressing Luc showed good sensitivity and excellent linearity by this optical imaging system. As explained in Fig. 1b, after linear fitting, an excellent correlation (coefficients of R^2^ > 0.99) was found between mKate2 and Luc expressing tumor cells. Moreover, since the slope of mKate2 is steeper than the one of Luc within 1 s, mKate2 expressing tumor performs higher sensitivity compared with Luc expressing tumor. Yet, compared with Luc expressing tumor, the fluorescence penetration ability of mKate2 is limited within 6 mm of imaging depth (Fig. 1c). In addition, before and after each experiment (including in vivo and in vitro experiments), tumor cells (Fig. 1d, e) and tumor tissues (Fig. 1f, g) were carefully examined under fluorescence microscope.

### Subcutaneous imaging

In order to investigate tumor development, as well as the report gene sensitivity in the subcutaneous compartment, mKate2 expressing tumor and a dual reporter gene Luc-GFP expressing tumor injected in the right and left flank of a single mice, respectively. Briefly, the first subcutaneous tumor images were taken 2 h post cell injection (Fig. 2a). At this time point particularly interesting was the intensity of mKate2 protein that was recorded around 1000 counts across the left to right side of the mouse (from 300 to 500 pixels). Based on preliminary data, the intensity of autofluorescence excited by 588 nm light was around 1000 counts in mice (n = 6) bearing an MDA-MB-435s-mKate2 tumor. These counts have been majorly derived from the autofluorescence contribution and, partly from filters’ crossover. Due to its reduced autofluorescence and the shallow position, the effective mKate2 fluorescence could be recorded without any complicated signal processing algorithms. However, the 1000 counts should be treated as negative signals and should be removed in the following study.

The rest of images were taken once a week for the next 30 days, Fig. 2b. The tumor burden (mm^2^) was calculated by quantification of tumor-luminescence surface area detected with the optical imaging system, Fig. 2e. Although bioluminescent Luc indicated great signal-to-noise ratio in vivo, mKate2 proteins perform comparable (Fig. 2c) or even better (Fig. 2d) signals under only 8 mW illumination within the bandwidth of 40 nm. Moreover, the exposure time of mKate2 was 30 times shorter, i.e. under current experimental conditions, the brightness of mKate2 was 30 times greater than the bioluminescence in the other cell line. Finally, after 4 weeks, the mouse was euthanized and the tumor tissue was examined (Fig. 2f). For the dual reporter gene Luc-GFP expressing tumor, GFP excitation and emission filters were used. This data suggests that both mKate2 fluorescent tumor and Luc bioluminescent tumor have the potential to be used for detection of subcutaneous tumor growth in vivo. Furthermore, mKate2 is considered a superior choice compared to Luc bioluminescence, because shorter exposure time is needed and no need of substrate injection.

### Abdominal imaging

To demonstrate the feasibility of the cell lines for in vivo imaging, mKate2 and Luc-GFP expressing tumor cells were also investigated in the abdominal region. Firstly, MDA-MB-435s-mKate2 cells were directly injected in the abdominal cavity. The first images (Fig. 3a), which were taken immediately after cell injection, indicated the location of tumor cells in the abdomen (position of injection). Three weeks later, 10 times greater fluorescence intensity was found in the central part of the abdomen which suggested that the tumor cell has proliferated and migrated to another location, (Fig. 3b–e), which was consistently confirmed by ex vivo examination (Fig. 3d). These results suggested that mKate2 could be used for tumor investigation in the abdominal area. However, unlike in the subcutaneous tumor case, diffusive photons can aggravate precise information over 1 mm in the living tissue, therefore fluorescent proteins in the diffusive regions of the living tissues were only able to approximately report the location of the tumor.

**Fig. 4:**
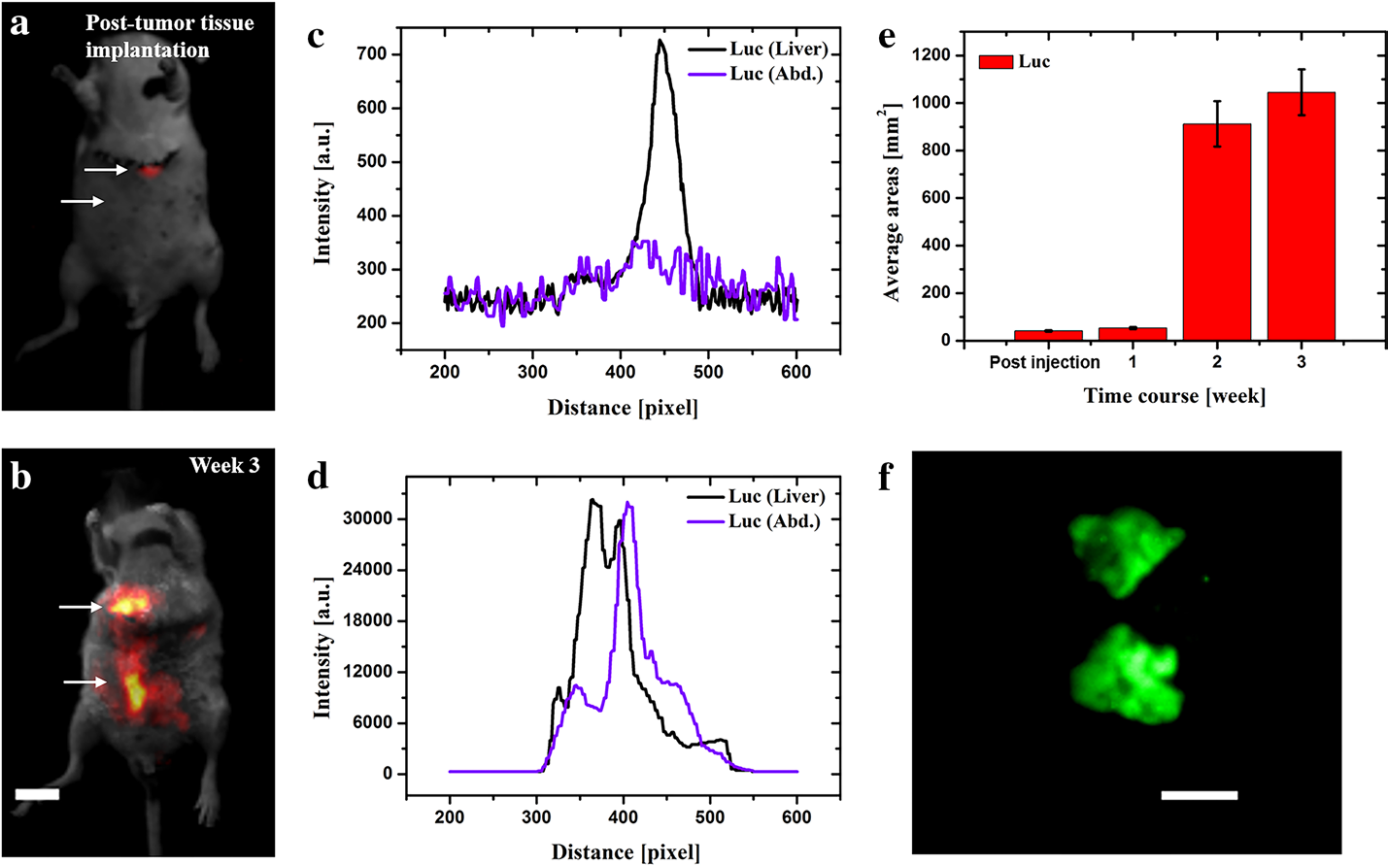
In vivo images of MDA-MB-231-Luc-GFP tumor tissue in the liver and abdominal area. **a**, **b** Images of tumor tissue implanted in the liver and, consequently tumor invasion and migration of cells in the abdominal cavity. **c**, **d** Intensity profiles are corresponding to the area along the arrow axis. Plot profiles of Luc in the liver and abdomen weeks are indicated in black and magenta, respectively. **e** Real-time quantification of total tumor-fluorescence surface area (mm^2^) detected over 3 weeks post-tumor tissue implantation. **f** Ex vivo tissue examination postmortem. Scale bar: 10 mm

In contrast to mKate2 expressing tumor, MDA-MB-231-Luc-GFP has shown to be a better choice when monitoring the tumor in a diffusive area such as abdominal cavity. Taking into account that bioluminescence report genes do not require an excitation light that consequently leads to significantly lower background (lower autofluorescence) for imaging, MDA-MB-231-Luc-GFP was tested using a different approach in order to simulate a fast and spontaneous tumor cells invasion. Briefly, 1 mm^3^ MDA-MB-231-Luc-GFP tumor tissue was carefully implanted in the liver (Fig. 4a). Over time, the tumor bioluminescence was detected with significantly higher intensity in the abdominal cavity, suggesting the rapid and spontaneous cell invasion in the abdomen (Fig. 4b, e). The corresponding intensity profiles of Fig. 4a, b are shown in Fig. 4c, d, respectively. Finally, the ex vivo results clearly presented the evidence of tumor tissue in the infected region (Fig. 4f). Furthermore, these results demonstrate that Luc reporter gene expressing tumor could be a good choice for studying a spontaneous tumor invasion and migration after further orthotropic tumor implantation in the liver.

### Deep tissue imaging

**Fig. 5:**
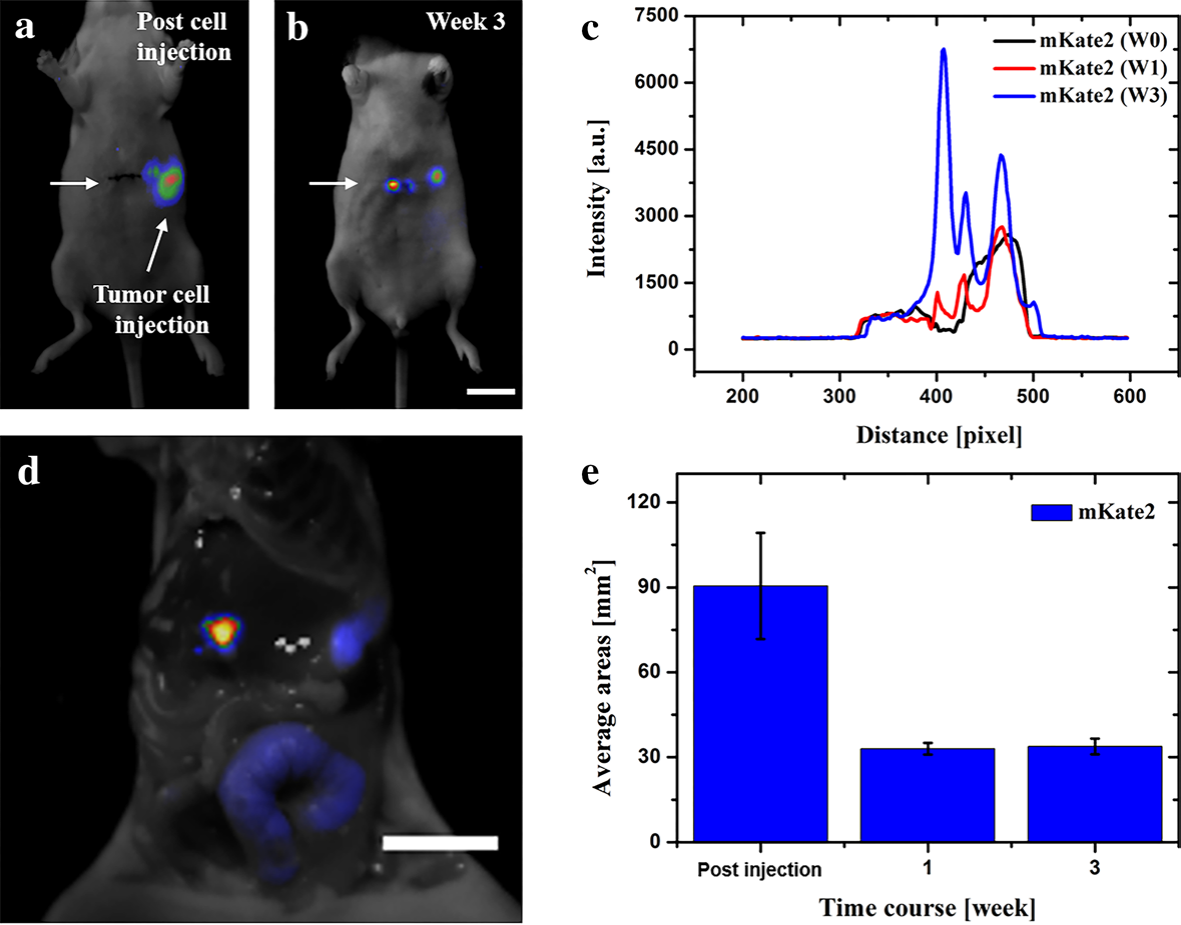
Fluorescence images of liver implanted MDA-MB-435s-mKate2 tumor cells. **a**, **b** In vivo images of tumor development in liver. **c** Intensity profiles are corresponding to the areas along the arrow axis. Plot profiles of mKate2 through different weeks are indicated in black, red and blue, respectively. **d** Ex vivo tissue examination postmortem. **e** Real-time quantification of total tumor-fluorescence surface area (mm^2^) detected over 3 weeks post-cells injection. Scale bar: 10 mm

Besides the diffusive media, complex circulation systems play a critical role when using in vivo imaging in deeper compartments such as liver. Oxygenated and deoxygenated hemoglobin are dominant photon absorbers in the visible spectrum region [38]. In the previous experiment, we have demonstrated that the Luc expressing tumor could be a very good choice for orthotropic tumor implantation in the liver because of low autofluorescence interfering from blood or other surrounding tissues. Yet, in order to avoid the existent fluorescent interference from hemoglobin, MDA-MB-435s-mKate2 cells were employed. Briefly, mKate2 expressing cells were injected directly in the left liver lobe. The first data was acquired immediately after surgical operation (Fig. 5a). Similar to the abdominal case, we noticed a rapid cell distribution in a large area of the liver. Consequently, after the wound healed completely, the fluorescence signal was detected with higher intensity, compared with week 1, in the central liver region (Fig. 5b). During the longitudinal observation of tumor growth, the lesion consolidated and proliferated suspiciously in the liver region (Fig. 5c, e). Once again, all suspicious areas were investigated ex vivo, confirming the mKate2 signal in the liver (Fig. 5d). These results have demonstrated that, besides bioluminescence, a far-red fluorescence protein mKate2 expressing tumor can also be used as a good choice for high blood organ imaging such as liver, with minimum interference detection from hemoglobin protein.

**Fig. 6:**
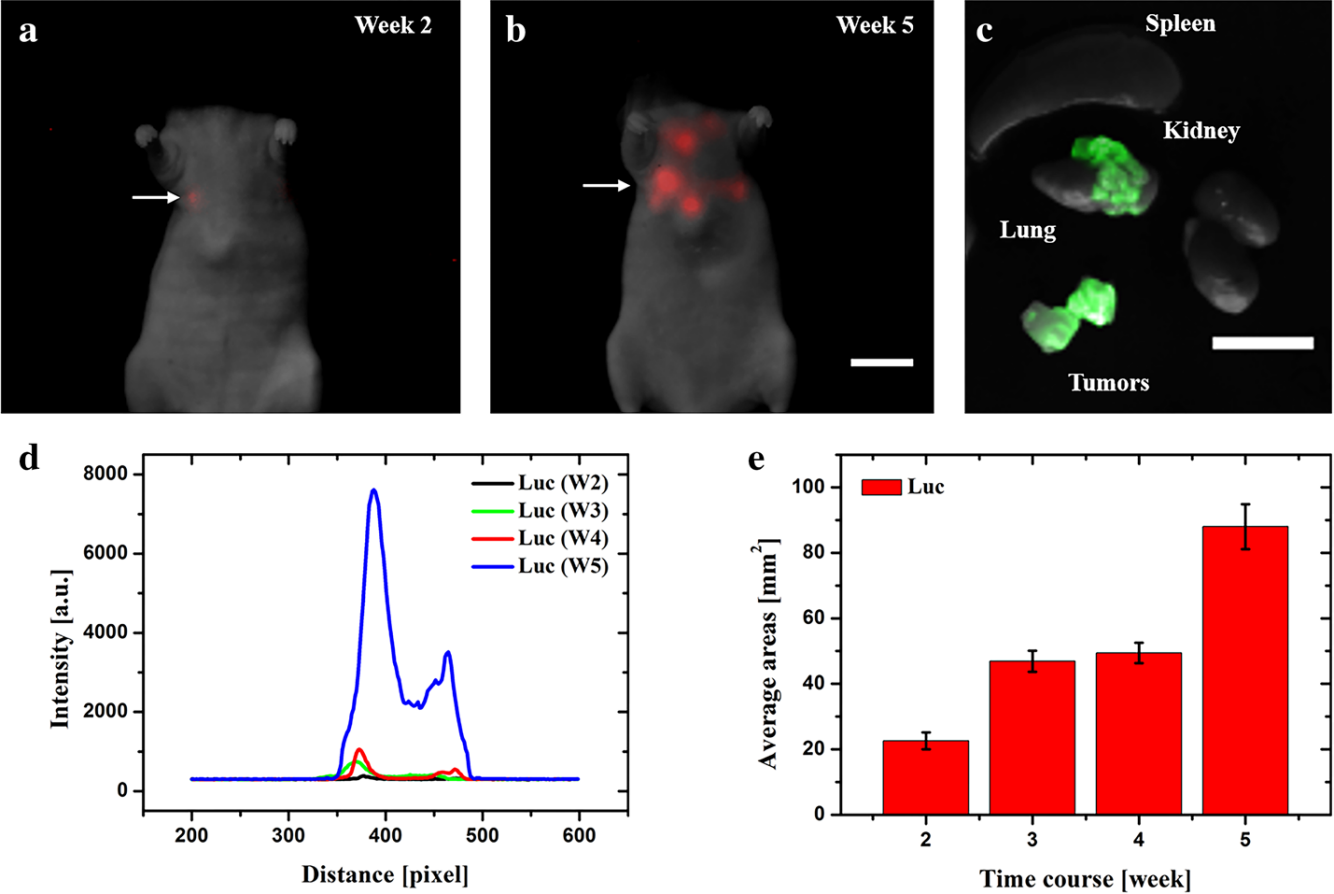
Investigation of MDA-MB-231 tumor expressing Luc-GFP in the mouse lungs. **a**, **b** In vivo images of tumor cells invading in the lung. **c** Ex vivo tissue examination postmortem. **d** Intensity profiles are corresponding to the area along the arrows. **e** T Real-time quantification of total tumor-fluorescence surface area (mm^2^) detected over 5 weeks post-cells injection. Scale bar: 10 mm

Finally, in order to investigate the Luc and mKate2 expressing tumor cells in even deeper body compartments such as lung, tail vein injection of the two cell lines were performed. Using this approach, tumor cells were allowed to spontaneously invade the mouse lungs. Two weeks post cell injection, bioluminescence signal was detected in the lung (Fig. 6a). In the following 3 weeks, other new sites of Luc signals were observed around the lung. These results indicated that the tumor invasion of a new lung region (Fig. 6b). The excellent signal to background ratio promised the long-term investigation of invasive and migratory conditions dynamically (Fig. 6d, e). After the mouse was dissected, the GFP fluorescence confirmed tumor lesions in the lung rather than other organs (Fig. 6c). Unfortunately, contrary to MDA-MB-231-Luc-GFP, we were not able to detect any mKate2 expressing tumor cells in that region, which may be explained from two aspects. The excitation and fluorescence light may not be able to penetrate through different intercostal space placed over the lungs. Besides, the excitation light could also contribute to the background. These results indicate that Luc reporter gene expressing tumor could be a good choice to trace the invasion and migration of tumor in deeper tissue, e.g. lung.

## Discussion

In this work, all data, were acquired by our home-made low-power imaging system. Our results suggested that this new system could be used for both fluorescence and bioluminescence imaging in live animals. Additionally, low power but sufficient excitation, is beneficial to alleviate the potential photo quenching and strong light induced cell damage [39, 40]. In the oncological studies, the far-red protein, mKate2, was useful to report tumor growth with high brightness, pH resistance and good photo stability no matter in subcutaneous tissues or in situ organs like liver. Over the past years, mKate2 has been applied to investigate mesenchymal stem cells (MSC) delivery by deep tissue imaging [41–43]. In some other studies, the reagents were inserted in the esophagus of euthanized mice [44, 45]. Moreover, mKate2 was used for whole body imaging after whole-body clearing [46]. Yet, so far, no studies have investigate the sensitivity and stability of mKate2 in deep tissue. The present study explored the application of mKate2 for real-time detection of tumor in whole-body, including the deep tissue compartment. Furthermore, to compensate the limited sensitivity of mKate2 in deeper body compartment such as lung, we also applied the firefly bioluminescent Luc to prove the feasibility of tracking the tumor proliferation, invasion and migration in those regions. The combination of bioluminescence and fluorescence reporters could be a highly valuable method for monitoring and detecting malignant cells in vivo. Based on anatomy of mice and other publications [47–49], the imaging depth of subcutaneous imaging could be 0.2–0.5 mm, the depth of abdominal imaging could between 0.5 and 1.0 mm, the depth of liver could be approximately 1 mm, and the depth of lung imaging could between 3 and 10 mm.

Currently, researchers tend to acquire three-dimensional information of location and size of tissue with relatively quantitative result, based on a reconstructive algorithm. This is indeed the future trend in this field, but this reconstructive algorithm has not yet reached a state of accuracy that allows its routine use in most labs worldwide. As a result, three-dimensional reconstruction requires longer time, while two-dimensional method could provide rapid information [50]. Our research group is simultaneously developing a new generation of optical tomography system and corresponding reconstructive algorithm for quantitative and three-dimensional study [51, 52]. The critical issues are the ill-posed nature of reconstruction and the acquirement of planar imaging with sufficient signal to noise ratio. For the first issue, researchers have already combined optical tomography with X-ray computed tomography together to get the precise priori information for reconstruction [53–55]. Moreover, on our multi-modality molecular imaging system [56], similar studies have been carried out. Moreover, even with priori information [57], researchers have already developed surface optical tomography system [58, 59]. Thus, in this work, we focused on the feasibility of using reporter genes for a long-term investigation of tumor growth in mice by a low-power imaging system, and in the future, this strategy holds the potential to be applied in these systems.

The autofluorescence in the epi-illumination system is one of the biggest challenges when working with fluorophores. Although, mKate2 showed very promising results in this work because of the reduced optical diffusion and increased wavelengths, autofluorescence still highly contributed with about 1000 counts to the background noise, which compressed the dynamic range of detector and deteriorated the detecting threshold in live tissues. In addition, skin and colon are the biological tissues that produce the majority of autofluorescence, thus limiting in vivo imaging quantification [60]. Furthermore, results from separate investigations on mKate2 and firefly Luc indicate that the combination of mKate2 and firefly Luc has the potential to compensate the performance of each other in different oncological studies in vivo; however, there should be a trade-off between exposure time and sensitivity. In order to acquire a better performance of genetic tagging tumor in deeper compartments, one direction is to use the far-infrared proteins [61–63]. With higher photostability and low cytotoxicity, these proteins can be applied for whole-body imaging. Recently, researchers have also studied time-resolved measurements, spectral un-mixing approaches and other methods based on subtraction of background [64–67]. All these methods have a potential to minimize the autofluorescence in this system in the future.

## Conclusions

In this paper, we demonstrated the feasibility and sensitivity of a near infrared (NIR) fluorescent protein mKate2 for a long-term non-invasive tumor imaging in live mice, by using a low-power optical imaging system (LP-OIS), which alleviates the photo quenching and cell damage induced by strong light. The combination of mKate2 and fLuc offers a superior choice for long-term non-invasive real-time investigation of tumor biological behavior in vivo.
